# Recruiting care homes to a randomised controlled trial

**DOI:** 10.1186/s13063-018-2915-x

**Published:** 2018-10-03

**Authors:** Alison Ellwood, Jennifer Airlie, Robert Cicero, Bonnie Cundill, David R Ellard, Amanda Farrin, Mary Godfrey, Liz Graham, John Green, Vicki McLellan, Najma Siddiqi, Anne Forster, On behalf of the REACH Programme Team, Karen Birch, David Ellard, Amanda Farrin, Joan Firth, Anne Forster, Bev Gallagher, Mary Godfrey, Elizabeth Graham, Claire Hulme, Rebecca Lawton, Najma Siddiqi, John Young

**Affiliations:** 1 Academic Unit of Elderly Care and Rehabilitation, Bradford Institute for Health Research, Bradford Teaching Hospitals NHS Foundation Trust, Temple Bank House, Bradford Royal Infirmary, Duckworth Lane, Bradford, West Yorkshire BD9 6RJ UK; 2 School of Biomedical Sciences, University of Leeds, Garstang Building, Leeds, LS2 9JT UK; 3 Clinical Trials Research Unit, Leeds Institute of Clinical Trials Research, University of Leeds, Level 10, Worsley Building, Clarendon Way, Leeds, LS2 9NL UK; 4 Warwick Clinical Trials Unit, Warwick Medical School, University of Warwick, Coventry, CV4 7AL UK; 5 Leeds Institute of Health Sciences, University of Leeds, Level 10, Worsley Building, Clarendon Way, Leeds, LS2 9NL UK; 6 Department of Health Sciences, Hull York Medical School, University of York, ARRC Building, Heslington, York, YO10 5DD UK

**Keywords:** Homes for the aged, Aged, Aged, 80 and over, Randomised controlled trials as topic, Research subjects, Vulnerable populations, United Kingdom, Recruitment, Care homes

## Abstract

**Background:**

There are more than a quarter of a million individuals aged ≥ 65 years who are resident in care homes in England and Wales. Care home residents have high levels of cognitive impairment, physical disability, multimorbidity and polypharmacy. Research is needed to ensure there are robust, evidence-based interventions to improve the quality of life of this frail group. However, there is a paucity of research studies in this area. Recruiting care homes and their residents to research is challenging.

A feasibility, cluster randomised controlled trial was undertaken as part of a research programme to identify ways to develop and test methods to enhance the physical activity of care home residents. This paper describes two methods of recruiting care homes to the trial and draws out learning to inform future studies.

**Methods:**

Eligible care homes met the following criteria: they were within a defined geographical area in the north of England; provided residential care for adults ≥ 65 years of age; had not previously been involved in the research programme; were not taking part in a conflicting study; were not recorded on the Care Quality Commission website as ‘inadequate’ or ‘requiring improvements’ in any area; and had ≥ 10 beds. Care homes were identified by a ‘systematic approach’ using the Care Quality Commission website database of care homes or a ‘targeted approach’ via a network of research-ready care homes. A standardised method was used to recruit care homes including eligibility screening; invitation letters; telephone contact; visits; formal letter of agreement.

**Results:**

In the systematic approach, 377 care homes were screened, 230 (61%) were initially eligible and invited to participate, 11 were recruited (recruitment rate (RR) 4.8%). In the targeted approach, 15 care homes were invited to participate, two were recruited (RR 13.3%). Overall, 245 care homes were approached and 13 recruited (RR 5.3%). A variety of care homes were recruited to the trial in terms of size, location, ownership and care provision.

**Conclusions:**

Systematic recruitment of care homes to the study was time-consuming and resource-heavy but led to a variety of care homes being recruited. The targeted approach led to a higher recruitment rate.

**Trial registration:**

ISRCTN registry, ISRCTN16076575. Registered on 25 June 2015.

## Background

Between 2005 and 2015, the UK population aged 65 years and over increased by 21%, and the population aged 85 years and over increased by 31% [1]. In 2011, 291,000 individuals aged 65 years and over lived in care homes (CHs) in England and Wales [2]. CHs ‘offer accommodation and personal care for people who may not be able to live independently. Some homes also offer care from qualified nurses or specialise in caring for particular groups’ [3], for example, people with dementia. With projected demographic changes the number of people living in CHs is likely to increase in the future [4]. CH residents are among the frailest of the population, distinguishable from community-dwelling older adults of the same age because of their dependency on others, cognitive impairment, multimorbidity and polypharmacy [5]. Accordingly, research specifically focused on the CH setting is necessary to try and address the challenges of health care for these vulnerable people and ensure robust, evidence-based service improvements are developed and implemented [6].

However, the CH setting has been largely neglected in research, particularly in clinical trials [7]. This could be partially attributed to the complexities and increased costs associated with recruiting vulnerable people, particularly those with cognitive impairment, to studies [8]. However, recruitment of vulnerable older adults to research has reported low decline rates, suggesting their willingness to be involved when given the opportunity [9, 10]. Whilst this is promising, recruiting CH residents to research presents particular challenges, including the need first to recruit CHs.

Recruiting CHs to research is also challenging. Access to CHs to conduct research requires the consent of managers and providers prior to approaching residents [11]. Unlike the National Health Service (NHS), the majority of CH providers are independent businesses offering a service for financial gain. CHs are often subject to scrutiny by the media, experience high staff turnover and must contend with pressurised situations, so allowing research teams to access their businesses may not be prioritised. Additionally, CH employees are often unfamiliar with research processes (e.g. randomisation, ‘blinding’ of researchers). Thus, studies undertaken in CHs may require greater researcher input when compared to similar work in hospitals or in the community [12]. It is likely that all these factors contribute to the paucity of involvement from the sector. In practice, this means that many CH studies are open to bias, as they are undertaken within homes preselected either by organisations [13] or through previous research involvement [10]. To address the problem of under-representation of CHs and their residents in high-quality studies, CH research networks (for example, Enabling Research in Care Homes — ENRICH) have been established [14, 15]. However, this may also result in potential bias by recruiting homes from a select group rather than offering involvement to a wider pool.

The Research Exploring Physical Activity in Care Homes (REACH) research programme sought to identify ways to develop and test methods to enhance the physical activity of CH residents. The final workstream of the programme was a feasibility, cluster randomised trial (CRT) of the REACH intervention plus usual care compared to usual care alone in preparation for a future definitive large-scale trial [16]. Data collection was at baseline and at 3, 6 and 9 months and required CH staff to collect data and to support residents with completion of outcome measures, including the wearing of an accelerometer on an elasticated belt around the waist and logging of time worn. Additionally, the intervention required a range of staff in the CH to be released to attend a series of three workshops and then to work with all the CH staff as a team to develop ways to encourage residents to improve their levels of movement. A systematic, rigorous and robust methodology was sought to give CHs within a defined geographical area the opportunity to participate in the trial, thereby decreasing selection bias. This paper describes the methods of recruiting CHs to the REACH feasibility CRT and the complexities encountered when recruiting CHs to involvement in a CRT. This process enabled a comparison to be made between two methods of recruiting CHs to the study: the systematic recruiting of CHs in a defined geographical area and recruitment via the local ENRICH network.

## Methods

CHs were eligible for the study if they were within a defined geographical area in the north of England, provided residential care (with or without additional specialist nursing and/or dementia care) for adults 65 years of age and over, had not been involved in previous REACH workstreams, were not taking part in a conflicting study or were not recorded on the Care Quality Commission (CQC) [3] website as ‘inadequate’ or ‘requiring improvements in any area’, as it was felt that the additional workload of a programme of enhanced care would have proved burdensome to homes addressing these concerns. In addition, for trial efficiency, it was agreed only to contact CHs with at least 10 beds to ensure that sufficient residents would be recruited to the study. In large multisite or multifloor establishments, one or two units within the home were eligible to be selected to participate as one home. The chosen units were identified by the home manager in discussion with the researcher.

The standardised method used by the research team for CH recruitment to the trial had four stages: 1. CHs were screened for eligibility and invitation letters were sent to eligible CHs; 2. CHs were contacted by telephone; 3. initial visits to the CH were undertaken; 4. interested CHs were given a letter of agreement to sign. There were three waves of recruitment using CHs identified through the CQC (labelled ‘systematic’ recruitment) and one using the ENRICH network (labelled ‘targeted’ recruitment). The plan was to recruit and randomise two CHs per month over a 6-month period.

### Systematic recruitment: first wave

#### Stage 1: screening for eligibility and invitation letters

In June 2015, CHs in West Yorkshire were identified using the publicly available care directory on the CQC website. This dataset was filtered to include only CHs providing residential care (with or without additional specialist nursing and/or dementia care) from the five local authority areas (population 2,226,058 in 2011 [17]), with 10 or more beds and which were categorised under the ‘older people’ service user band. All CHs identified were individually screened using the eligibility criteria. Eligible CHs were then sent an information pack by post inviting them to consider participation in the REACH feasibility trial. The pack included an introductory letter, information sheet about the study and a reply slip with which to register interest or to decline participation.

#### Stage 2: telephone contact

On receipt of reply slips registering interest in the study, researchers re-checked that the CHs were not ineligible (i.e. they were exclusively a nursing home or provided care for younger people) and their current status on the CQC website. The CHs were then telephoned to confirm their eligibility, to discuss the likely resource requirements for participating (CH staff assisting with data collection and, depending on randomisation, possible involvement in the intervention) and to confirm they had no current involvement in any other conflicting research trials or studies.

Attempts were made to telephone CH managers who did not respond to the initial letter about the study after first having checked on the CQC website that their status had not changed and that they remained open and provided residential care for older people. Eligibility criteria were confirmed when contact was made with these CHs. Those expressing interest were visited as described in the following section.

#### Stage 3: initial visits

Meetings were arranged with the managers of CHs still interested in being involved following the initial telephone conversation. Further details about the study, time commitments, staff roles and trial processes were provided at this meeting, and any queries CH managers had were answered.

#### Stage 4: letter of agreement

CHs agreeing to participate were asked to sign a formal letter of agreement between both the CH manager and owner and the research sponsor (Bradford Teaching Hospitals NHS Foundation Trust) to enable them to participate in the study. The letter of agreement set out the duties and responsibilities of both parties, including agreement to allow access to the CH for the REACH research team for the purposes of data collection over four specified periods and agreement to make a modest payment of £600 to the CH for continued involvement in the study.

Following the poor response rate to the first wave of recruitment outlined above, two further recruitment waves via the CQC database were made. These are outlined in the following text.

### Systematic recruitment: second wave

In January 2016, CHs initially deemed as ineligible due to their CQC status were re-screened. CHs now re-rated as ‘good’ or requiring improvements in only one area of the CQC report were invited to participate, using the process outlined in the first wave as previously described.

### Systematic recruitment: third wave

In February 2016, the initial screening area was widened, and residential CHs from specific areas of North Yorkshire were identified (using the same CQC directory from stage 1), screened and approached using the same recruitment process as outlined previously for the first wave. As with the second wave, homes were included if they required improvements in only one area. ENRICH homes already approached were excluded (see the following section).

### Targeted recruitment through the ENRICH network

In January 2016, the Yorkshire Enabling Research in Care Homes (ENRICH) network assisted with contacting CHs in their network within specified areas of North Yorkshire. Invitations were emailed to each CH with details on how to express interest in participating in the study. Details of CHs expressing interest in the study were passed on to researchers to establish eligibility and to contact and arrange visits as outlined previously for the first wave.

Researchers kept contemporaneous notes of all visits and contacts with CHs.

## Results

**Fig. 1:**
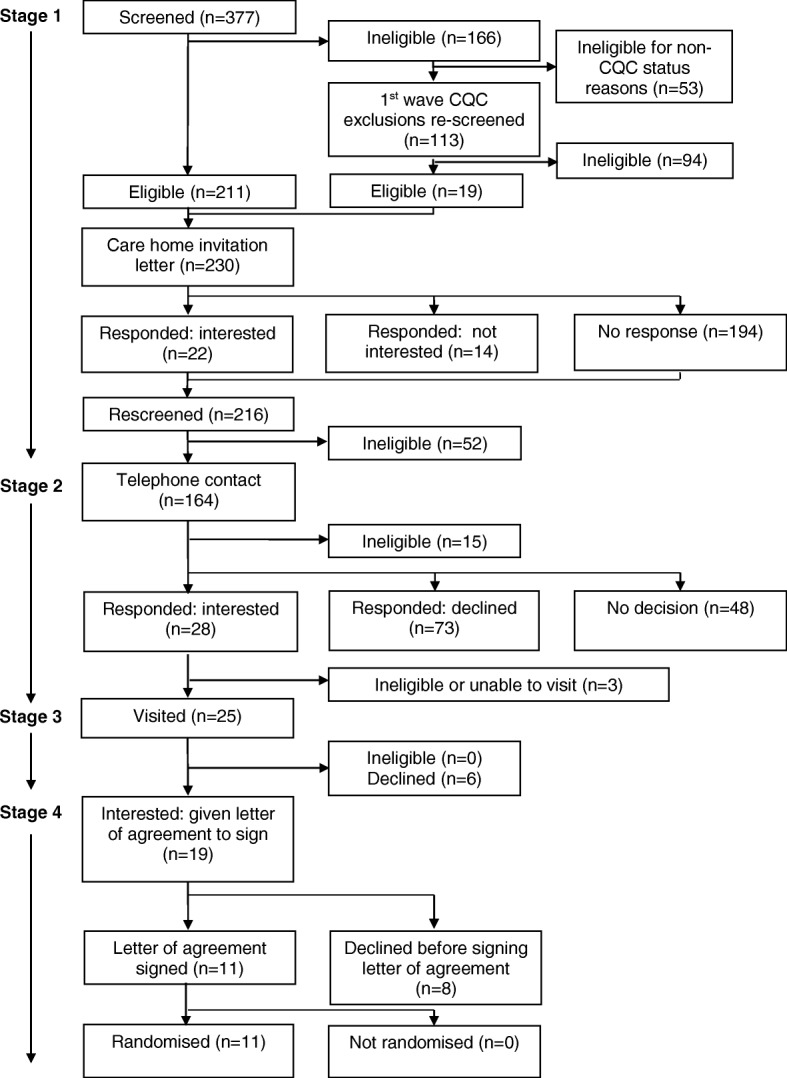
Consort diagram: care home recruitment flow chart for first to third waves (‘systematic’ recruitment)

**Fig. 2:**
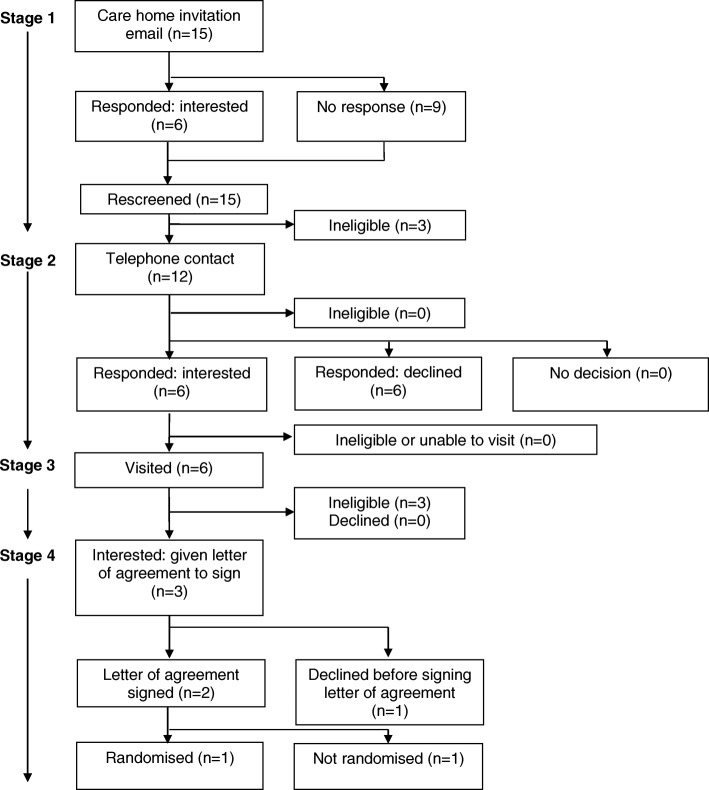
Consort diagram: care home recruitment flow chart for ‘targeted’ recruitment via the ENRICH network

CH screening commenced in June 2015, the final CH letter of agreement was signed in July 2016 and the final CH was randomised in September 2016. The recruitment target of 12 CHs was achieved in 16 months. This required the equivalent of approximately one full-time researcher. Figures 1 and 2 are Consolidated Standards of Reporting Trials (CONSORT) diagrams showing respectively the flow of CHs through the recruitment processes for systematic recruitment via the CQC databases (the first to third waves) and targeted recruitment via the ENRICH network.

### Stage 1: CH screening and invitation letter

#### Systematic recruitment: first to third waves

Three hundred and seventy-seven CHs were screened, and 147 (39.0%) were excluded. Two hundred and thirty CHs were invited to participate in the study. Of the 36 CHs (15.7%) responding to the invitation letter, 22 (9.6%) expressed interest in the study: 61% of those responding. These 22 interested CHs, and the 194 CHs not responding to the invitation letter, were re-screened before an attempt was made to contact them via telephone. Fifty-two were found to be ineligible despite being initially eligible at the first screening, due to changes in CQC status, closures and ineligible service provision.

#### Targeted recruitment through the ENRICH network

Fifteen CHs in the ENRICH network were emailed by a senior community research nurse from the Yorkshire and Humber Clinical Research Network, based upon location but not screened for eligibility, and invited to participate in the study. Six CHs (40%) responded to the invitation email, all expressing interest in the study. A further nine CHs not responding to the invitation email were re-screened before an attempt was made to contact them via telephone. Three were found to be ineligible, due to changes in CQC status and ineligible service provision.

### Stage 2: CH telephone contact

Telephone contact was made with 164 eligible CHs in the first to third waves and 12 of the ENRICH network CHs. Of these, 15 (all systematic approach CHs) were deemed ineligible, 34 (28 systematic and 6 targeted) expressed (or maintained) interest and 79 (73 systematic and 6 targeted) declined, typically citing ‘busyness’ or lack of interest in research as the reason (Table 1). The remaining 48 CHs (all systematic) had initially expressed interest and requested further information via email, post or follow-up calls. However, this interest was not maintained despite the continued efforts of the research team. Three of the 34 interested CHs contacted by telephone expressed interest, but a visit could not be arranged or they were later found to be ineligible.

**Table 1:** Reasons for non-participation of care homes at telephone contact stage

Reason	Systematic (*n* = 73)	Targeted (*n* = 6)	Total (*n* = 79)
Too busy	20 (27.4%)	0 (0%)	20 (25.3%)
Unable to contact	14 (19.2%)	0 (0%)	14 (17.7%)
Not interested in research	11 (15.1%)	0 (0%)	11 (13.9%)
Unstable management or home	11 (15.1%)	0 (0%)	11 (13.9%)
Participation in research initiated only through head office	4 (5.5%)	0 (0%)	4 (5.1%)
Disapproves of research	1 (1.4%)	0 (0%)	1 (1.3%)
No reason given	10 (13.7%)	0 (0%)	10 (12.7%)
Missing data	2 (2.7%)	6 (100.0%)	8 (10.1%)

### Stage 3: CH meetings

The research team visited 31 of the 34 interested CHs (25 systematic and 6 targeted) 47 times to discuss the study: systematic (median (interquartile range [IQR]; range)) visits: 2 (1–2; 1–3); targeted: 1 visit each CH. Of the 31 CHs visited, three were found to be ineligible (all targeted ENRICH CHs), and six CHs declined participation (all systematic sites). The remaining 22 CHs (19 systematic and 3 targeted) were given a letter of agreement to sign.

### Stage 4: letter of agreement

A letter of agreement was provided to 22 CHs (19 systematic and 3 targeted). Despite repeated contact from the research team, nine did not sign the letter of agreement (8 systematic CHs; 1 targeted CH). Five managers, including the targeted site, declined due to difficulties engaging their head offices in the project, and four experienced a change in circumstances affecting their capacity fully to engage with the project. Thirteen CHs signed the letter of agreement, and 12 CHs were subsequently randomised; one of these was a targeted CH. The remaining CH (a targeted home) was not randomised because the required sample size had then been reached.

The overall recruitment rate was 5.3% (13 CHs recruited from 245 approached by post or email). The recruitment rate for the systematic approach was lower than that of the targeted approach: 4.8% (11 CHs recruited from 230 approached) versus 13.3% (2 CHs recruited from 15 approached) respectively.

The average time taken to recruit the CHs (from initial visit to gaining access to screen residents for eligibility) varied. In the systematic approach CHs, this ranged from 7 to 134 days (median (IQR) 56 (32.25–92.75) days); of the two targeted approach CHs recruited, only one was randomised, and the time between initial visit and access to residents for data collection was 97 days. The time taken to recruit the independently owned CHs was less than for the CHs run by larger organisations (median (IQR) days: 48 (17–59) versus 80 (54–131) respectively).

A variety of CHs were randomised to the REACH trial in terms of size, location, ownership and care provision (Table 2). Units from three CHs were recruited rather than the whole CH. Nine CHs (all systematic) were classified as small/medium (≤ 40 beds) [15], and four as large (> 40 beds). The median (IQR; range) of beds in the CHs or units was 29 (18–37.5; 12–64) beds. Six CHs were located in suburban locations, four in semi-rural locations, two in urban locations and one in a rural area (the CH recruited through the ENRICH network). Five CHs were independently owned, three were part of not-for-profit organisations, four were part of a ‘chain’ and one was owned by a local authority. In addition to residential care, one CH provided nursing care and one CH provided respite care. The three CHs where units were involved additionally provided specialist residential dementia care.

**Table 2:** Characteristics of recruited care homes

	Systematic (*n* = 11)	Targeted (*n* = 2)	Total (*n* = 13)
Size			
Small/medium	9 (81.8%)	0 (0%)	9 (69.2%)
Large	2 (18.2%)	2 (100.0%)	4 (30.8%)
Recruited component			
Care home	9 (81.8%)	1 (50.0%)	10 (76.9%)
Unit	2 (18.2%)	1 (50.0%)	3 (23.1%)
Number of beds in care home or unit			
Mean (SD)	28 (10.26)	41 (32.53)	30 (14.13)
Median (IQR)	29 (18–35)	41	29 (18–37.5)
Range	12–44	18–64	12–64
Location			
Urban	2 (18.2%)	0 (0%)	2 (15.4%)
Suburban	6 (54.5%)	0 (0%)	6 (46.2%)
Semi-rural	3 (27.3%)	1 (50.0%)	4 (30.8%)
Rural	0 (0%)	1 (50.0%)	1 (7.7%)
Ownership			
Independent	5 (45.5%)	0 (0%)	5 (38.5%)
Chain	2 (18.2%)	2 (100.0%)	4 (30.8%)
Not-for-profit	3 (27.3%)	0 (0%)	3 (23.1%)
Local authority	1 (9.1%)	0 (0%)	1 (7.7%)
Provision of care in care home or unit			
Residential	8 (72.7%)	0 (0%)	8 (61.5%)
Residential, dementia	2 (18.2%)	1 (50%)	3 (23.1%)
Residential, nursing	1 (9.1%)	0 (0%)	1 (7.7%)
Residential, dementia, respite	0 (0%)	1 (50%)	1 (7.7%)

*SD* standard deviation, *IRQ* interquartile range

## Discussion

A systematic, standardised and rigorous procedure was adopted to recruit CHs to a feasibility CRT in a defined geographical area to assess the suitability of this method of recruitment for a large-scale definitive trial. This was supplemented by targeted recruitment using a similar standardised method. A range of CHs were recruited in terms of size, location, ownership and provision (Table 2). However, recruitment proved to be time-consuming and resource-heavy: it took approximately 16 months from the start of the initial screening to randomising the final CH and approximately the equivalent of one full-time researcher recruiting CHs via either approach. In order to recruit the target of 12 CHs, 245 CHs were approached by post or email. The recruitment rates of 5.3% overall and 4.8% for the systematic approach and 13.3% for the targeted approach are far lower than others reported (27–73%) [14]. However, many studies do not explicitly describe how CHs were recruited. It may be they sourced ‘research-ready’ CHs and did not approach all eligible CHs within a geographical area.

There was a poor response by CHs (16%) to the first invitation letter (first to third waves of systematic recruitment). Many CHs subsequently explained that they did not recall seeing the information or had been too busy to respond to it. Targeted recruitment proved more successful: out of 15 ENRICH network CHs emailed, six (40%) responded to the invitation. However, given that CHs in the ENRICH network are ‘research-ready’, this response was disappointing.

Concurrent changes to the CQC inspection process led to many CHs requiring some improvements. It was noted that minor improvements required to medication administration procedures and deprivation of liberty safeguarding documentation were frequently recorded on CQC reports. On review, these were not deemed by the research team, which included colleagues with experience in CH work, as likely to affect their capacity to undertake the REACH research project or intervention. CHs requiring improvements in one area are still rated as 'good' for the purposes of CQC auditing. The decision was therefore made to relax the initial inclusion criterion relating to CQC status from 'no improvements needed' to 'one improvement needed' in a second and a third wave of screening.

Given the continual state of flux of the CH sector, repeated checking of CH eligibility was deemed necessary throughout the recruitment process. This time-consuming but essential process was repeated each time a CH was telephoned: of the CHs re-screened prior to telephone contact, 55 (24%) were found to be ineligible or excluded despite being initially eligible at the first screening. This also has implications for the conduct of trials, particularly those that are of any significant duration, as homes that were initially eligible may subsequently become ineligible in a short space of time.

Maintaining the interest of CHs required frequent contact from the researchers. The changeable and distinctive CH settings required flexibility, sensitivity and extensive planning from the researcher, who was experienced in CH work, including the timing of visits, adapting to last-minute cancellations and an awareness of the low priority given to research. Provision of CH information packs was helpful in the meetings with CH managers, many of whom had had little experience with such research.

The burdens of data collection and the requirements of the intervention on their staff were important considerations for managers in deciding whether to participate or not. For some, the staff time necessary to support data collection by researchers was reckoned to be too great. A number of managers also remarked they would participate in studies requiring less of their time, e.g. completion of questionnaires.

Managers also balanced the requirements of the intervention on their staff with what they perceived as free ‘training’. Indeed, some were particularly keen to receive the ‘training’ despite being repeatedly informed of the random allocation to intervention or usual care. A number of CH managers may have declined because the intervention and associated training did not accord with mandatory training or fit with existing practice.

Obtaining signatures from both the CH manager and owner on a letter of agreement, a formal requirement for CH participation, was usually straightforward in independently run private businesses but, despite CH managers being keen to be involved in research [11], was often problematic and time-consuming in larger CH organisations. Indeed, in some cases, these delays caused eventual withdrawal of interest from homes [14]. Despite being part of a research network, there was little evidence that this process was more straightforward or expedited in the targeted CHs, where obtaining head office signatures encountered the same delays as for the systematic CHs. A subsequent reduction in the length and complexity of the letter of agreement appeared to expedite the sign-off in the later stages of CH recruitment. The position large corporations took regarding research also affected uptake. A number of CHs indicated that they would only take on research projects signposted to them by their head office, or in some cases they had research programmes of their own.

To sustain CHs’ interest in the study, protracted and repeated contacts (face-to-face meetings and telephone calls) were often necessary between the initial expression of interest and researchers gaining access to the CH to screen residents. More visits were undertaken to the systematic approach CHs than to the targeted approach CHs. This was more a reflection of their location further away from the researcher base rather than their being part of the ENRICH network. Interest was most difficult to sustain in the period after the letter of agreement had been provided to CHs but had not been signed by the owner. This was less difficult, however, in independent CHs, where the owner was usually more readily available.

Intervention studies in CHs, especially those requiring input from the whole home (staff, residents and management) are uncommon. Previous research has typically involved collection of data from databases and records or from resident or staff interviews or observational work [18, 19], which was consonant with the views expressed by some CH managers. Development of the ENRICH network reflects both the need of studies such as REACH and the difficulty in attracting CHs to involvement in research. Although development of such networks is largely positive, their use may limit access to other CHs for the purpose of research, leading to a biased sample. Utilisation of the ENRICH network for this study yielded greater interest than mailshotting CHs; however, only two homes of the 15 approached signed the letter of agreement (13.3%). This suggests that additional work could be done by the ENRICH network to improve CH staff members’ understanding of the variety of research projects in which they may be able to engage.

Previous REACH workstreams and other studies utilised a more ad hoc method of recruiting CHs via forums, previous contact, ‘snowballing’ or approaching the head offices of large chains [20, 21]. This may have required less researcher time. However, the aim of the present study was to establish a systematic, rigorous and robust methodology to recruit a representative and unbiased sample of CHs, capable of replication in a future large-scale trial. Although this methodology reduces bias, recruited CHs were still self-selecting and subject to eligibility criteria, which limited involvement. This may have an impact on generalisability of findings if only interested and ‘good’ homes are involved. Involving CHs, which are usually private businesses and run for financial gain, will carry a risk of bias towards those homes which practise an open-door policy and welcome involvement from external parties. Using CQC status as an eligibility criterion excluded access to the study for many CHs in the geographical area. Whilst the CQC status of a number of CHs changed during involvement with REACH, engagement by managers and staff with the requirements of the research in these homes was largely consistent over time, perhaps calling into question the purpose of restricting approach only to homes with a ‘good’ rating. It is possible that CHs may utilise involvement in research as evidence of steps taken to improve service provision. Potential change of status meant that this eligibility criterion may therefore be both unstable and an unreliable means of selecting CHs to approach.

The recruitment method utilised two sources of potential CHs: a systematic approach via a comprehensive database of CHs accessed through the CQC website, and a targeted approach via a network of research-ready care homes (ENRICH). Although only two CHs were recruited via the ENRICH network, the recruitment rate was higher than that obtained via the systematic approach using the CQC database (13.3% versus 4.8%), which suggests a less labour-intensive method of CH recruitment. However, as only two CHs were recruited via the ENRICH network, and given the small numbers of CHs approached, it is not known whether using this method more widely would result in a broad range of CHs being recruited.

A strength of the study was a rigorous, systematic unbiased method of recruitment, which led to a broad range of CHs being recruited, as shown in Table 2. This method should also allow for larger scale recruitment in a full-scale trial. However, this method is costly and time-consuming in terms of researcher hours, and may have lengthened the time required to secure CHs to the study. This systematic method was supplemented by a targeted method of recruitment through a network of research-ready CHs. Requirements for eligibility affected the number of CHs with research experience, as REACH eligibility excluded those currently involved in other trials or conflicting studies. Increasing the number of CHs that are familiar with research promotes knowledge and understanding of the processes and benefits for residents and staff.

## Conclusions

A significant amount of time needed to be apportioned for the recruitment of the CHs to the REACH study. This needs to be taken into account in terms of time and budget when designing similar CH research, both in terms of the length of engagement required (i.e. a realistic timeline) and the actual hours assigned to researchers for contacting managers to secure and maintain their interest.

Streamlining the processes of recruitment (i.e. simplifying the letter of agreement for CHs’ head office approval required to access the CH) increased and expedited recruitment. Gaining the approval of larger corporations prior to approaching CHs may be a method of increasing recruitment and expediting access to the homes. However this must be balanced against a potential for sample bias if the head office purposively select homes. A targeted approach (for example via a network of research-ready CHs) may lead to a greater recruitment rate, but due to the small numbers recruited in this study it is not known whether this would lead to a diversity of CHs being recruited.

## Abbreviations

CH: Care home
CONSORT: Consolidated Standards of Reporting Trials
CQC: Care Quality Commission
CRT: Cluster randomised trial
CTRU: Clinical Trials Research Unit
ENRICH: Enabling Research in Care Homes
NHS: National Health Service
NIHR: National Institute for Health Research
REACH: Research Exploring Physical Activity in Care Homes
